# Interplay of Inflammatory Mediators with Epigenetics and Cartilage Modifications in Osteoarthritis

**DOI:** 10.3389/fbioe.2018.00022

**Published:** 2018-03-14

**Authors:** Swarna Raman, Una FitzGerald, J. Mary Murphy

**Affiliations:** ^1^Orthobiology, Regenerative Medicine Institute, National University of Ireland Galway, Galway, Ireland; ^2^School of Natural Sciences, National University of Ireland Galway, Galway, Ireland

**Keywords:** inflammation, chondrocytes, osteoarthritis, epigenetics, cartilage, methylation, microRNA, hypertrophy

## Abstract

Osteoarthritis (OA), a degenerative disease of diarthrodial joints, is influenced by mechanical and inflammatory factors with aging, obesity, chronic injuries, and secondary diseases thought to be major factors driving the process of articular cartilage degeneration. Chondrocytes, the cellular component of cartilage, reside in an avascular environment and normally have limited potential to replicate. However, extrinsic factors such as injury to the joint or intrinsic alterations to the chondrocytes themselves can lead to an altered phenotype and development of OA. Synovial inflammation is also a pivotal element of the osteoarthritic, degenerative process: influx of pro-inflammatory cytokines and production of matrix metalloproteinases accelerate advanced cellular processes such as synovitis and cartilage damage. As well as a genetic input, recent data have highlighted epigenetic factors as contributing to disease. Studies conducted over the last decade have focused on three key aspects in OA; inflammation and the immune response, genome-wide association studies that have identified important genes undergoing epigenetic modifications, and finally how chondrocytes transform in their function during development and disease. Data highlighted here have identified critical inflammatory genes involved in OA and how these factors impact chondrocyte hypertrophy in the disease. This review also addresses key inflammatory factors in synovial inflammation, epigenetics, and chondrocyte fate, and how agents that inhibit epigenetic mechanisms like DNA methylation and histone modifications could aid in development of long-term treatment strategies for the disease.

## Introduction

Osteoarthritis (OA) is the most common musculoskeletal disorder impacting life quality of patients with about 35–40 million Europeans and ~355 million affected worldwide (Barry and Murphy, 2013; Mobasheri, 2013). With the disease predominantly affecting the aging population, the impact of OA is set to increase exponentially in coming decades (Kim et al., 2015). Understanding of OA has progressed from what was regarded as a condition affecting articular cartilage to the concept that the disease process has broader consequences affecting all joint tissues, including the synovium and ligaments as well as the underlying subchondral bone (Gupta et al., 2012; Barry and Murphy, 2013) and is characterized as a whole-joint disease. OA is also a multifaceted disease based on recent findings that, apart from cellular and molecular mechanisms, inflammation, metabolic processes, and epigenetic modifications (Shen et al., 2017) are involved in the pathogenesis and progression of the disease (Blanco and Rego-Perez, 2014; Ishijima et al., 2014; Courties et al., 2015).

## Cartilage and Associated Components

Articular cartilage is a highly specialized connective tissue that ensures normal function and optimal load-bearing capacity (Sophia Fox et al., 2009). It coordinates with the synovial membrane and associated synovial fluid to bring about frictionless movement. The components of articular cartilage are the extracellular matrix (ECM) with sparsely distributed specialized cells called chondrocytes, collagen fibers (mainly type II and IX), proteoglycans, water, and a small volume of non-collagenous proteins and glycoproteins such as fibronectin (Sophia Fox et al., 2009). Chondrocytes are indispensable for the development, maintenance, and repair of the cartilaginous ECM. They are highly specialized, non-proliferating cells with adult articular cartilage being an avascular tissue in nature (Goldring et al., 2011; den Hollander et al., 2015). Under normal conditions, permanent hyaline cartilage found in articulating joints does not undergo terminal differentiation (van der Kraan and van den Berg, 2012). However, based on *in vitro* and *in vivo* evidence, re-programming of articular chondrocytes toward a hypertrophic, degradative phenotype frequently occurs as OA develops (van der Kraan and van den Berg, 2007, 2012). OA chondrocytes behave like terminally differentiated chondrocytes in the growth plate with processes such as aging, biomechanical stress, inflammation, and altered methylation status triggering abnormal phenotypic changes with active production of metalloproteinases, particularly matrix metalloproteinases (MMP)-13 for example (van der Kraan and van den Berg, 2012).

With respect to the collagen network, the complex triple helical structure of the collagen type II α-polypeptide chains along with associated collagenous and non-collagenous matrix proteins provides vital sheer and tensile properties to stabilize the matrix (Goldring, 2000a,b). Aggrecan (ACAN) is the most abundant proteoglycan, followed by decorin, biglycan, and fibromodulin. The interaction of ACAN with hyaluronan through link protein to form proteoglycan aggregates is optimal for resisting compressive loads (Sophia Fox et al., 2009). Other associated matrix components playing a vital role in cartilage structure and function in association with the collagen network include, small leucine-rich proteoglycans, like decorin, biglycan, fibromodulin, and lumican (Heinegard et al., 2006). Therefore, in OA, local loss of proteoglycan and cleavage of type II collagen at the cartilage surface results in an influx of water content and loss of tensile strength in the cartilage ECM matrix as lesions progress (Goldring, 2000a,b). Cartilage matrix disruption in OA is usually associated with altered chondrocyte behavior and clustering, which in turn changes the composition of matrix components (Goldring, 2000a,b, Goldring and Otero, 2011). Several studies conducted both *in vitro* and *in vivo* have proved the involvement of pro-inflammatory cytokines and metalloproteinases in matrix disruption. These factors mainly target chondrocytes, causing aberrant expression of catabolic and anabolic genes. Of the matrix-degrading enzymes produced by hypertrophic chondrocytes, MMP-13 is critical in its ability to cleave type II collagen with cleavage products shown to produce OA-like effects in the mouse knee joint (Neuhold et al., 2001; Goldring et al., 2011).

## Synovial Inflammation and OA

The synovial membrane is vital for maintenance of articular cartilage and the health of the joint as a whole. It functions to (1) secrete synovial fluid for joint lubrication with hyaluronic acid the principal component that provides the required viscosity; (2) supplement nutrition to chondrocytes and draining waste metabolites, and (3) act as the source of synoviocytes, macrophages, and fibroblasts that express a wide range of cellular markers required for specialized functions relating to innate and adaptive immunity (Hettinga, 1979; Scanzello and Goldring, 2012). Inflammation of the synovium, or synovitis, is associated with OA and is seen in both early and late OA (Benito et al., 2005; Sohn et al., 2012). It is believed that inflammation is one of the major causes for acceleration of cartilage destruction and ultimately disease progression (Egloff et al., 2012). Synovitis has also been documented with meniscal injury leading to OA and is associated with pain and dysfunction (Berenbaum, 2013). Synovitis is known to directly influence many clinical symptoms including knee effusion, redness, heat, and swelling (Ayral et al., 1999; Sellam and Berenbaum, 2010). Ultrasonography and MRI have also depicted synovitis in OA and the afore-mentioned symptoms and synovitis have been associated with the radiographic progression of OA (Ledingham et al., 1995; Iagnocco and Coari, 2000; Benito et al., 2005; Loeuille et al., 2005). Microarray and gene pattern analysis of synovial tissues from patients without radiographic evidence of OA undergoing arthroscopic meniscectomy showed that 43% of patients had synovial inflammation that correlated with traumatic meniscal injury and pain. Synovial biopsy specimens that showed high inflammation scores, recorded a strong chemokine signature, involving significant levels of chemokine ligand-5 (CCL5), CCL7, CCL19, and interleukin-8 (IL-8) (Scanzello et al., 2011).

Synovial inflammation is classically characterized by influx of macrophages and T cells, increased neovascularization, and subsequent secretion of pro-inflammatory cytokines (Sellam and Berenbaum, 2010). The impact of synovitis throughout disease progression was demonstrated by Benito et al. who compared key immunohistological features of inflammation during early and late OA. Immunohistochemical staining of synovial tissue samples with gross synovial hypertrophy showed high expression of markers of inflammatory cell infiltration (CD4^+^ and CD68^+^) angiogenesis with increased levels of vascular endothelial growth factor (VEGF), blood vessel formation (factor VIII), and intercellular adhesion molecule-1 and the pro-inflammatory cytokines, tumor necrosis factor-α (TNF-α), and IL1ß in both early and late OA, with significantly higher expression in early OA (Benito et al., 2005). Analysis of proteins in the synovial fluid collected from patients with knee OA showed the presence of 108 different proteins that included plasma proteins, serine protease inhibitors, proteins pertaining to cartilage turnover, and proteins involved in inflammation and immunity (Sohn et al., 2012). In addition to this, Sohn et al., demonstrated higher levels of pro-inflammatory cytokines like TNF-α, interleukin-6 (IL-6), and VEGF in OA synovial fluid compared to healthy individuals. Analysis of stimulated macrophages derived from TLR-4 deficient and wild-type mice proved that plasma proteins and inflammatory cytokines present in synovial fluid indeed function as damage-associated molecular patterns signaling through TLRs to mediate an early response to injury and damage to the joint (Scanzello et al., 2008; Midwood et al., 2009; Sohn et al., 2012).

## Epigenetic Mechanisms and OA

The term “epigenetics” can be explained as heritable modifications to gene expression/transcription, without altering the underlying DNA sequence (Blanco and Rego-Perez, 2014; Zhang et al., 2015). Mechanisms of epigenetic regulation documented in OA pathogenesis include DNA methylation, histone modification, and non-coding RNAs (Simon and Jeffries, 2017) (Zhang et al., 2015). DNA methylation has been documented in OA as both hypo and hypermethylation that generally occurs in promoter CpG sites of target genes (Im and Choi, 2013). Methylation, ubiquitination, acetylation, sumoylation, and phosphorylation are some of the histone modifications, with methylation and acetylation documented as the most recurrent histone changes in OA (Blanco and Rego-Perez, 2014; Kim et al., 2015). Short ncRNAs such as microRNAs (miRNAs) act as catalytic or regulatory RNAs by binding to specific sites in the 3′-untranslated region of target mRNAs, resulting in mRNA degradation and/or inhibition of translation (Zhang and Wang, 2015; Ramos and Meulenbelt, 2017).

**Figure 1:**
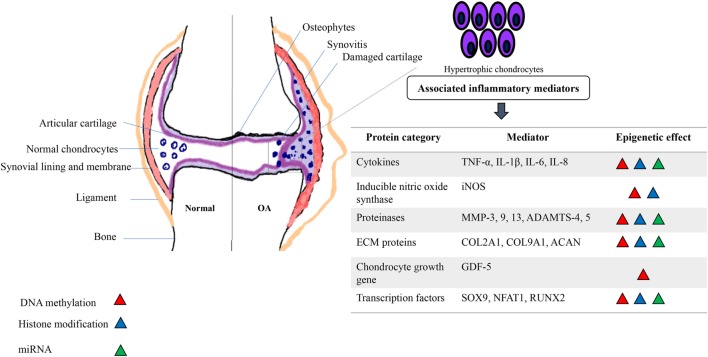
Schematic depicting the association of inflammatory mediators and epigenetic modifications in an osteoarthritic joint. A pathological loop is created due to constant release of cartilage degrading proteins and cytokines and aberrant epigenetic modifications further alter the phenotype and function of normal adult chondrocytes, pushing toward a “hypertrophic state” causing further damage to the joint.

Therefore, like all somatic cells, normal functional adult articular chondrocytes are subjected to epigenetic mechanisms that aid in stabilizing their phenotype. However, certain innate environmental interactions can cause epigenetic changes in chondrocyte gene expression, which are passed on to daughter cells in subsequent divisions (Goldring and Otero, 2011). This generates “modified/altered chondrocytes,” with respect to their phenotype and function that is associated with overexpression of cartilage-degrading proteases and inflammatory mediators (Blanco and Rego-Perez, 2014). This process culminates in disruption of cellular homeostasis, causing cartilage ECM degradation and a constant pathological loop involving inflammation and epigenetic modifications resulting in expedited disease progression (Figure 1). There is no doubt that OA has a strong genetic component (Valdes and Spector, 2011; Barter et al., 2012; Blanco and Rego-Perez, 2014). However, “low penetrance polymorphisms” in the population, partly due to inheritance of epigenetic modifications, is a reason for limited data generation to aid in the identification of genes responsible for the genetic susceptibility to OA (Valdes and Spector, 2011; Barter et al., 2012). In the last decade or so, candidate gene studies and genome-wide approaches have shown how inflammatory genes are modulated by epigenetic modifications [reviewed in Rogers et al. (2015)]. These data link specific inflammatory mediators, including transcription factors, proteinases, cytokines, chemokines, growth factors, and ECM proteins with a well-defined role of epigenetic changes in OA and induction of synovial inflammation. Interleukin-1 beta (IL-1β) is a classic example of a pro-inflammatory cytokine involved in the immune-pathogenesis of OA that is epigenetically regulated (Goldring et al., 2011; Reynard and Loughlin, 2012) and promotes inflammation of the synovium (Scanzello and Goldring, 2012). It is seen to be elevated in the synovial fluid of OA patients (Sandy et al., 2015) and micro-array analysis of IL-1 gene expression showed that *IL1R*, encoding the IL-1 receptor, is also upregulated in OA (Rogers et al., 2015). In addition to effects on synovial inflammation, epigenetic control of these mediators is also associated with chondrocyte fate toward cartilage destruction and hypertrophic chondrocyte formation.

## Inflammatory Mediators and Epigenetic Mechanisms

### Cytokines

Cytokines secreted by immune cells have been known to have pleotropic effects in models of rheumatoid arthritis (RA) for many years (Feldmann et al., 1996). Inflammation is also a driving factor in disease progression in OA with aberrant expression of cytokines a major cause in both animal and human models of the disease (Goldring and Otero, 2011). Among the cytokines studied in OA, tumor necrosis factor-alpha (TNF-α) and interleukin-1 beta (IL-1β) are two of the most extensively researched pro-inflammatory cytokines with respect to their gene activation, methylation status, and their indirect effects on proteinases like MMP-13 (Table 1) (Goldring, 2000a,b; Hashimoto et al., 2009, 2013; Kapoor et al., 2011).

**Table 1:** Critical genes associated with chondrocyte fate and synovial inflammation.

Critical genes	Protein category	Association	Epigenetic mechanism	Reference
IL-1β	Cytokine	Synovium and chondrocyte alteration	DNA methylation, microRNA (miRNA) (34a, 140a, 146a) histone modification	Hashimoto et al. (2009, 2013); Yamasaki et al. (2009)
Tumor necrosis factor-α	Cytokine	Synovium and chondrocyte alteration	miRNA (149)	Zhang and Wang (2015)
Interleukin-8	Cytokine	Synovium and chondrocyte hypertrophy	DNA methylation	Takahashi et al. (2015)
Interleukin-6	Cytokine	Synovium	DNA methylation and histone modification	Yang et al. (2017)
Nitric oxide (NO)	Isoform of NO synthase release- NF-κB pathway	Synovium and chondrocyte hypertrophy	DNA methylation, histone modification	de Andrés et al. (2013)
Matrix metalloproteinases (MMP)-3	Proteinase	Chondrocyte alteration	DNA methylation	Roach et al. (2005)
MMP-9	Proteinase	Chondrocyte alteration	DNA methylation	Roach et al. (2005)
MMP-13	Proteinase	Synovium and chondrocyte alteration	DNA methylation, miRNA (22, 27a, 27b, 146a) histone modification	Roach et al. (2005); Higashiyama et al. (2010)
ADAMTS-4	Proteinase	Chondrocyte alteration	DNA methylation	Roach et al. (2005); Hashimoto et al. (2009)
ADAMTS-5	Proteinase	Synovium	miRNA (140a, 27a, 27b, 146a)	Goldring and Marcu (2012); Ukai et al. (2012)
COL2A1	Extracellular matrix (ECM) protein	Chondrocyte alteration	Histone modification, miRNA (34a, 675)	Tsuda et al. (2003); Goldring and Marcu (2012); Ukai et al. (2012)
COL9A1	ECM protein	Chondrocyte alteration	DNA methylation	Imagawa et al. (2014)
ACAN	ECM protein	Chondrocyte alteration	Histone modification, miRNA (337)	Pöschl et al. (2005); Huh et al. (2007); Hong et al. (2009); Ukai et al. (2012); Zhong et al. (2012); Im and Choi (2013)
GDF-5	Growth and differentiation factor	Synovium and chondrocyte alteration	DNA methylation(hypo)	Miyamoto et al. (2007); Southam et al. (2007); Egli et al. (2009)
RUNX-2	Transcription factor	Chondrocyte alteration	DNA methylation	Wang et al. (2004); Kamekura et al. (2006); Hecht et al. (2007); Higashikawa et al. (2009)
NFAT1	Transcription factor	Chondrocyte alteration	Histone modification	Rodova et al. (2011); Zhang and Wang (2015)
SOX-9	Transcription factor	Chondrocyte alteration	DNA methylation, miRNA (574-3p), histone modification	Martinez-Sanchez et al. (2012); Kim et al. (2013)

Hashimoto et al. studied the epigenetic regulation of IL-1β expression in OA cartilage by analyzing CpG sites in the IL-1β promoter and identified that methylation of CpG site-299 modulated promoter activity by suppression of transcriptional function (Hashimoto et al., 2013). In another study conducted by the same authors, demethylation of the same CpG site in the IL-1β promoter of human articular chondrocytes resulted in elevated transcription of other inflammatory cytokines in response to treatment by IL-1β itself (Hashimoto et al., 2009). In addition to being epigenetically regulated, IL-1β also impacts miRNA production by chondrocytes (Akhtar et al., 2010). Among the miRNAs studied in association with pro-inflammatory cytokines, miRs 140, 149, and 146a have shown a prominent association with human articular cartilage homeostasis, OA pathogenesis, and chondrocyte alteration (Yamasaki et al., 2009; Li et al., 2011; Zhang and Wang, 2015). miR146a was shown to be expressed in low-grade OA with significantly increased expression seen after stimulation with IL-1β (Yamasaki et al., 2009). Downregulation of miR149 in OA chondrocytes revealed its modulatory effect in the actual production of TNF-α, IL-1β, and IL-6 (Santini et al., 2014). IL-1β signaling also modulated the expression of 909 out of 3,459 genes in primary human articular chondrocytes in addition to induction of IL11 and CCL-5 (Saas et al., 2006; Rogers et al., 2015).

The impact of IL-1β expression *via* its promoter activity and the capacity of this cytokine in stimulating other cartilage degrading proteases can be interrogated through the use of histone modification and histone deacetylase inhibitors (HDACi). The process of acetylation is orchestrated by histone acetyltransferases, located on specific lysine residues on the histone N-terminal tails, resulting in the unraveling of the histone and providing access to the DNA structure and transcriptional machinery (Clayton et al., 2006; Barter et al., 2012). HDAC activity is critical for the maintenance of chondrocyte phenotype (Huh et al., 2007) and based on previous reports, HDAC 1 and 2 are upregulated in OA chondrocytes (Hong et al., 2009). Depletion of HDAC7 was also found to be directly proportional to MMP-13 expression (Higashiyama et al., 2010). HDACi have been used in models of RA (Chung et al., 2003) and OA (Chen et al., 2010) to investigate the catabolic activity of HDAC in chondrocytes, and the use of HDACi has elicited beneficial effects by suppressing synovitis, reducing secretion of inflammatory cytokines (particularly IL-1) and preventing the redifferentiation of dedifferentiated chondrocytes (Huh et al., 2007).

Elevation of IL-1β can cause an influx of nitric oxide (NO) (de Andrés et al., 2013), a short-lived, multi-functional inflammatory molecule, expressed in the form of the inducible isoform of NO synthase (iNOS) (Table 1) (Charles et al., 1993). However, addition of HDACi, trichostatin, and butyric acid to IL-β-stimulated human chondrocytes suppressed the expression of iNOS and prostaglandin E2 and prevented IL-1-induced proteoglycan release from cartilage explants (Chabane et al., 2008). This clearly depicts the effect of HDACs on IL-1 and in turn on chondrocyte function in OA; further research into HDACi inhibitors could provide directions toward treatment of OA by targeting aberrant epigenetic modifications. Expression of iNOS can also be altered by DNA methylation. When human chondrocytes were co-transfected with nuclear factor kappa-light-chain-enhancer of activated B cells (NF-κB) subunit p65 and an enhancer element carrying the NO gene with or without p50, demethylation of 6 out of 7 CpG sites in the iNOS promoter element was documented, confirming the effect of methylation in iNOS induction (de Andrés et al., 2013).

Interleukin-8, also called CXCL-8, is a more recent inflammatory chemokine to be studied in the context of OA. Alongside IL-1β and leukemia inhibitory factor, IL-8 induces chondrocyte hypertrophy and differentiation (Borzì et al., 1999; Borzi et al., 2002; Merz et al., 2003; Takahashi et al., 2015). In a study conducted by Pierzchala et al. (2011), synovial fluid from OA patients showed significantly increased levels of IL-8 compared to controls (Takahashi et al., 2015). Demethylation of CpG sites in the IL-8 promoter resulted in a 37-fold higher gene expression in cultured chondrocytes from OA patients. On the other hand, *in vitro* DNA methylation reduced basal activity of the IL-8 promoter (Table 1) (Takahashi et al., 2015). IL-6 is a macrophage-derived pro-inflammatory cytokine seen in high levels in synovial fibroblasts (SF), with DNA hypo-methylation and histone hyper-acetylation influencing its overexpression in OA. Yang et al. showed that SF from OA patients had significant DNA hypo-methylation at three specific CpG sites in the IL-6 promoter. Similarly, these cells showed increased H3K9/K14 and H4K12 acetylation in the IL-6 promoter region compared to SF from non-arthritic donors. Furthermore, treatment of OA SF with DNA methyltransferase 3 alpha or anacardic acid, a histone acetyltransferase inhibitor, resulted in increased DNA methylation and decreased histone acetylation, leading to a suppression in IL-6 overexpression (Yang et al., 2017). These studies clearly show the effect of epigenetic mechanisms of cytokines and their subsequent expression during OA and determination of chondrocyte fate.

### Transcription Factors

Transcription factors, also known to regulate chondrocyte differentiation, act by controlling the transcriptional rate of target genes and aberrant expression is known to influence OA pathogenesis as a consequence of chondrocyte hypertrophy (van der Kraan and van den Berg, 2012; Zhang and Wang, 2015). Some of the key factors involved in altering chondrocyte fate include Runt-related transcription factor 2 (Runx2), nuclear factor of activated T cells 1 (Nfat1), sex determining region Y-Box 9 (SOX9) (Table 1), and hypoxia-inducible factor-2alpha (HIF-2alpha) (van der Kraan and van den Berg, 2012). Recent genome-wide analysis showed that demethylation of 5mC to 5hmC was associated with dynamic expression changes in expression of Runx2 and SOX9 during mouse embryonic skeletal development and early and late chondrogenic differentiation (Taylor et al., 2016). Runx2 controls chondrogenic differentiation, specifically, chondrocyte hypertrophic differentiation, with evidence of elevated expression seen in human OA cartilage and many experimental models (Wang et al., 2004; Kamekura et al., 2006; Hecht et al., 2007; Higashikawa et al., 2009).

NFAT1 is a well-known regulator of expression of cytokine genes during the immune response, and interestingly, exhibits an age-dependent expression in mouse articular cartilage (Zhang and Wang, 2015). Rodova et al. conducted a study assessing the effect of histone methylation in Nfat1 expression in wild-type and Nfat1-deficient mice. Nfat1 expression in wild-type articular chondrocytes was seen to be low during embryonic stages and increased in adult mice. As a consequence of histone methylation, embryonic articular chondrocytes showed increased Nfat1 expression with a simultaneous increase in H3K4me2, a histone associated with transcriptional activation. However, decreased expression of Nfat1 in 6-month-old articular chondrocytes, correlated with increased H3K9me2 and transcriptional repression, suggestive of the crucial role played by histone methylation in age-related Nfat1 expression (Table 1) (Rodova et al., 2011; Zhang and Wang, 2015). This indeed provides the rationale for further study to identify how Nfat1 expression is influenced by epigenetics modifications in human OA.

A key transcription factor for chondrogenesis during the development of skeletal system, SOX9 is one of the earliest markers expressed by mesenchymal stem/stromal cells and is essential for expression of cartilage-specific matrix proteins (Han and Lefebvre, 2008; Zhang and Wang, 2015). Kim et al. (2013) reported the downregulation of SOX9 expression in late stage hip OA chondrocytes due to DNA and histone methylation and histone acetylation. Elevated miR145 in human chondrocytes is also associated with direct repression of SOX9 expression (Martinez-Sanchez et al., 2012).

## Key OA Risk Gene-Growth and Differentiation Factor 5 (GDF5)

Among many genes subjected to candidate gene association analysis (Ryder et al., 2008) for OA, GDF5 has shown consistent association with OA in the form of a single nucleotide polymorphism rs143383, located in the 5’ untranslated region of the gene (Table 1) (Chapman et al., 2008). GDF5, an important member of TGF-β superfamily and an extracellular signaling molecule, influences the development, maintenance, and repair of the synovial joint and tissue structures (Khan et al., 2007). Allelic transition from C to T methylation of the associated CpG dinucleotide results in a differential allelic expression (DAE) imbalance, thereby affecting joint tissue. In OA patient joint tissues examined, reduction in GDF5 transcription was found to be a result of a functional rs143383, with the T allele mediating this effect. Additionally, this effect was seen in both older and younger cartilage samples, indicative of a chronic and deeper involvement of DAE imbalance in OA (Miyamoto et al., 2007; Southam et al., 2007; Egli et al., 2009). A similar effect of rs143383 was seen in a mouse model, where a reduction in GDF5 mRNA and protein, recapitulated an OA-like phenotype (Daans et al., 2011). Hence, maintenance of GDF5 protein has been found to be critical for normal joint function (Reynard and Loughlin, 2012).

### ECM Proteins

Maintaining optimal amounts of ECM components, such as collagen and proteoglycan, is also crucial for preserving normal articular cartilage architecture and function. Gene mutations of collagen proteins are typically associated with early-onset of OA (Chan et al., 1995). For example, collagen type IX (COL9A1)-deficient mice portray OA-like cartilage degradation (Table 1) (Fässler et al., 1994; Saamanen et al., 2000). Histone modifications are also known to affect type-II collagen (COL2A1) gene expression in OA. Histone acetylation at the COL2A1 promoter enhanced its transcription activity due to complex formation of SOX9 with the HAT p300/CBP in chondrocytes (Tsuda et al., 2003; Ramos and Meulenbelt, 2017).

Interactions of HDACi with chondrocytes have conflicting effects on COL2A1, COL9A1, and ACAN gene expression. Short-term HDACi treatment of chondrocytes enhances the expression of these genes, whereas long-term treatment represses their expression. This switch in effects could be attributed to overexpression of HDAC1 and 2 (Table 1) (Huh et al., 2007; Hong et al., 2009; Im and Choi, 2013). Hypermethylation at CpG sites in the COL9A1 promoter attenuated SOX9 binding to COL9A1, leading to downregulation of the collagen in OA cartilage (Imagawa et al., 2014). DNA methylation does not influence the CpG sites in the ACAN promoter, unlike HDACs that control ACAN in both normal aged and OA chondrocytes (Pöschl et al., 2005) and miR199a-3p and 193b in human OA chondrocytes (Ukai et al., 2012).

### Proteinases

Normal human articular cartilage expresses very low levels of matrix proteinases such as aggrecanases, collagenases, and MMPs with elevated levels in OA associated with ECM degradation and altered chondrocyte phenotype (Burrage et al., 2006; Huang and Wu, 2008). Aggrecanases, ADAMTS (a disintegrin and metalloproteinase with thrombospondin motifs) -4 and -5, and MMPs-3, 9, and 13 are the most prominent proteinases involved in chondrocyte hypertrophy and inflammation with DNA methylation and histone modifications involved in their control (Table 1) (Roach et al., 2005; Hashimoto et al., 2009; Ramos and Meulenbelt, 2017). Alteration in chondrocyte phenotype due to changed gene expression is not seen in all OA chondrocytes, but specifically in those proximal to weight-bearing regions and in the surface zone (Roach and Tilley, 2007; Hashimoto et al., 2009). Previous studies have documented elevated expression of MMP-3, 9, 13, and ADAMTS-4 in late-stage OA chondrocytes as a result of hypomethylation of promoter CpG sites (Roach et al., 2005; Cheung et al., 2009; da Silva et al., 2009).

MicroRNAs have a strong role to play in the regulation of MMPs and aggrecanases. ADAMTS-5 expression is highly regulated by miRNA in human OA (Ukai et al., 2012). In a study conducted by Young et al., comparing cultured SW1353 chondrosarcoma cells and primary human chondrocytes, addition of HDACi downregulated the expression of MMP-13, a critical marker of chondrocyte hypertrophy (Higashiyama et al., 2010). In fact, many studies have demonstrated how miR-140, 27a, and 29a directly target MMP-13 (Tardif et al., 2009; Li et al., 2016). These data expand the possibilities for targeted OA therapy by focusing on miRNA regulation in chondrocyte fate and function.

## Conclusion

Osteoarthritis is indeed a multifactorial disease underpinned by a complex interplay of inflammation and epigenetic modifications. Genome-wide analyses and studies focused on the identification of candidate OA-risk associated genes conducted in the last decade have thrown light on the mechanism of how chondrocytes undergo hypertrophy and cause progression of the disease. Future studies targeting markers involved in both chondrocyte and synovial hypertrophy could perhaps provide further insight into the use of epigenetic knockdown models and inhibitors to attenuate chondrocyte terminal differentiation and in turn restore cartilage homeostasis.

## Author Contributions

SR contributed to the development of the concept underpinning the review, researched the relevant literature, and wrote the body of the review. UF also contributed to the concept of the submission and provided guidance on the scientific writing. MM participated in conception and review design, manuscript preparation, on-going and final review of the submission.

## Conflict of Interest Statement

The authors declare that the research was conducted in the absence of any commercial or financial relationships that could be construed as a potential conflict of interest.
